# Impact of Deep Learning 3D CT Super-Resolution on AI-Based Pulmonary Nodule Characterization

**DOI:** 10.3390/tomography11020013

**Published:** 2025-01-27

**Authors:** Dongok Kim, Chulkyun Ahn, Jong Hyo Kim

**Affiliations:** 1 Department of Applied Bioengineering, Graduate School of Convergence Science and Technology, Seoul National University, Seoul 08826, Republic of Korea; dongk@snu.ac.kr; 2 ClariPi Research, ClariPi Inc., Seoul 03088, Republic of Korea; rnd3456@claripi.com; 3 Department of Radiology, Seoul National University Hospital and College of Medicine, Seoul 03080, Republic of Korea; 4 Center for Medical-IT Convergence Technology Research, Advanced Institutes of Convergence Technology, Suwon 16229, Republic of Korea

**Keywords:** deep learning, computed tomography, lung nodules, super-resolution, slice thickness

## Abstract

Background/Objectives: Correct pulmonary nodule volumetry and categorization is paramount for accurate diagnosis in lung cancer screening programs. CT scanners with slice thicknesses of multiple millimetres are still common worldwide, and slice thickness has an adverse effect on the accuracy of the pulmonary nodule volumetry. Methods: We propose a deep learning based super-resolution technique to generate thin-slice CT images from thick-slice CT images. Analysis of the lung nodule volumetry and categorization accuracy was performed using commercially available AI-based lung cancer screening software. Results: The accuracy of pulmonary nodule categorization increased from 72.7 percent to 94.5 percent when thick-slice CT images were converted to generated-thin-slice CT images. Conclusions: Applying the super-resolution-based slice generation on thick-slice CT images prior to automatic nodule evaluation significantly increases the accuracy of pulmonary nodule volumetry and corresponding pulmonary nodule category.

## 1. Introduction

National lung screening programs initiated in Europe have increased the demand for chest computed tomography (CT) scans, and when it comes to pulmonary nodule volumetry, slice thickness in CT images is a key factor [1]. Unfortunately, not all CT scans are taken with uniform thin-slice thickness, and therefore a method to neutralize the effect of heterogeneous slice thickness is desired. Here, we introduce a deep learning-based super-resolution method to convert thick-slice CT images to generated-thin-slice CT images.

Advancements in deep learning, particularly in super-resolution techniques, have significantly surpassed traditional interpolation methods in image quality restoration. Dong et al. [2] pioneered one of the earliest super-resolution methods using a three-layer convolutional neural network (CNN), achieving superior results compared to conventional interpolation techniques. However, the shallow depth of the network constrained its ability to effectively recover high-frequency image details. To address this limitation, Kim et al. [3,4] incorporated ResNet principles, designing a 32-layer CNN with skip connections to mitigate the vanishing gradient problem. Zhang et al. [5] further refined the approach by introducing a residual-in-residual structure, resulting in high peak signal-to-noise ratio (PSNR) and structural similarity index (SSIM) values.

Ledig et al. [6] highlighted the limitations of relying solely on the L1 loss function, which tends to produce blurred outputs by prioritizing averaged pixel values and sacrificing high-frequency details. Additionally, PSNR and SSIM metrics do not always correlate well with perceived image quality. To address these issues, they proposed a perceptual loss based on feature maps from a pre-trained VGG network [7] and utilized a generative adversarial network (GAN) [8] for training. In this framework, the generator created super-resolved (SR) images from low-resolution (LR) inputs, while the discriminator differentiated between SR images and high-resolution (HR) ground truths. This adversarial training enabled the generator to produce images nearly indistinguishable from HR images, leading to the development of a Super-resolution Generative Adversarial Network (SRGAN). Although SRGAN did not achieve the highest PSNR and SSIM scores, it generated SR images with perceptual quality closer to HR images.

Based on SRGAN, Wang et al. [9] introduced Enhanced SRGAN (ESRGAN), incorporating residual-in-residual dense blocks without batch normalization, which further improved image quality. Subsequently, Real-ESRGAN [10] added degradations such as noise, blur, and JPEG compression during training to enhance robustness for real-world applications.

In the medical imaging domain, researchers have actively adapted deep learning-enabled super-resolution techniques to advance the field. Park et al. [11,12,13] utilized residual networks to up-sample in the z-direction and generate thin CT slices, leveraging this technology to explore radiomic features in chest CT. More specifically, they explored whether SR methods improve radiomic feature reproducibility across varying CT slice thicknesses. A total dataset of one hundred series of CT images from lung cancer patients with 1 mm, 3 mm and 5 mm slices thicknesses were used to train the model. The developed model was then used to convert 3 mm and 5 mm thick slices to 1 mm thin slices. Radiomic reproducibility decreased with increased slice thickness, but the SR technique standardized radiomic feature extraction and improved diagnostic consistency.

Yun et al. [14,15] generated thin CT slices to achieve higher quality orbital bone reconstructions. They identified critical issues in the field of 3D orbital bone reconstruction, which were the aliasing effects and structural disconnections caused by thick CT slice thickness. The orbital bone, especially its thin structures like the medial wall and orbital floor, is prone to fractures, but thick-slice CT images fail to accurately represent these fine structures, hampering the creation of precise 3D models for surgical implants. To convert thick slices to thin slices, orbital bone edge-aware loss was implemented where it specifically targeted the orbital bone region using a mask that identified bone edges, ensuring the preservation of thin and cortical bone structures. The network architecture had a 2D CNN with six convolutional layers for feature extraction and up-sampling layers for inter-slice resolution enhancement. Skip connections between the layers maintained low-level features, such as textures and gradients, crucial for retaining edge details. The proposed network offered a robust solution for improving inter-slice resolution in facial CT imaging, particularly for orbital bone reconstruction.

Nakamoto et al. [16] also showed a method used to generate thin-slice CT images from thicker-slice CT images. Their virtual thin-slice technique used a conditional generative adversarial network framework, comprising an encoder–decoder-style generator inspired by U-Net and a discriminator designed for 3D data processing. The generator employed skip connections to retain spatial information, enabling the reconstruction of high-resolution thin-slice CT images from thick-slice CT inputs. It used 3D convolutional layers to handle the volumetric nature of CT data, optimizing the output through adversarial loss, which promoted realistic image generation, and L1 loss, which minimized pixel-wise intensity differences between generated and true thin-slice images. The discriminator, also built with 3D convolutional layers, distinguished real thin-slice images from generated ones and incorporated conditioning labels, such as slice intervals, to improve the accuracy of the super-resolution process. Together, the generator and discriminator were trained to produce isotropic thin-slice images with a voxel size of 1 × 1 × 1 mm, ensuring high fidelity and realistic morphology for volumetric CT image reconstruction. This enhanced the visibility of anatomical details in CT images, particularly in high-contrast regions like bone. However, they concluded that it may have had diagnostic limitations for subtle abnormalities, highlighting the need for further refinement, due to the tendency of GANs to normalize slight deviations, such as mild compression fractures, which could affect diagnostic utility.

Iwano et al. [17,18] used same model to show how the measurement of solid size in early-stage lung adenocarcinoma affected in thick-slice CT images and virtual 3D thin-slice CT images. They concluded that the conversion of thick-slice CT images to thin-slice CT images improved the detection and measurement of pulmonary nodules, thereby increasing staging accuracy and possibly providing better prognosis prediction.

Building upon these advancements, we propose a deep learning-based three-dimensional (3D) super-resolution method to generate thin-slice CT images from heterogeneous thick-slice CT images. Following on, a commercially available AI-based lung cancer screening software (ClariPulmo, v2.0.0, ClariPi, Seoul, Republic of Korea) [19] was utilized to evaluate the consistency of pulmonary nodule categorization by measuring pulmonary nodule volumes and classifying the nodules in accordance with well-established Lung-RADS v2022 published by the American College of Radiology on thick-, generated-thin- and thin-slice CT images.

## 2. Materials and Methods

### 2.1. Training Dataset

For training the deep learning model for CT slice generation, we used the National Lung Screening Trial (NLST) open dataset. CT images with slice thicknesses of 1.0 mm were selected, which consisted of 18,243 images from the Siemens Sensation 16 scanner, 2931 images from the Canon Aquilion scanner, 2707 images from the GE LightSpeed 16, scanner and 4747 images from the Philips MX8000 scanner, resulting in a total of 28,628 chest CT images (Table 1).

### 2.2. Test Dataset

**Figure 1:**
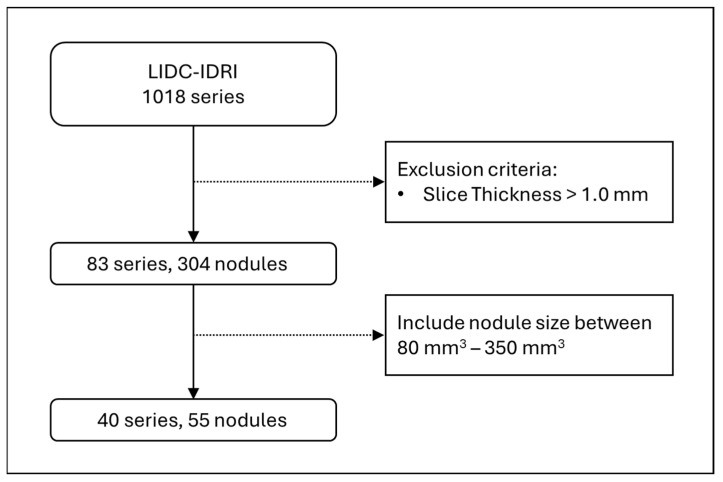
A flow diagram of pulmonary nodule inclusion for the test dataset.

For testing the deep learning model for CT slice generation, we used the Lung Image Database Consortium and Image Database Resource Initiative (LIDC-IDRI) open dataset. LIDC-IDRI contains 1018 series, and among them we selected thin-slice CT images which had slice thicknesses of 1.0 mm or less, and that resulted in 83 series and 304 nodules. Lung-RADS v2022 categorises pulmonary nodules between 2, 3, 4A, and 4B, based on its solid volume and total volume, and those volumes of interest mostly range between 80 and 350 mm^3^. Therefore, we further filtered the nodule sizes to be in that range, which left us with a total of 40 series and 55 nodules to be used as the test dataset (Figure 1).

### 2.3. Model Architecture

The base of our super-resolution framework was influenced by ESRGAN, with necessary modification made to suit medical imaging applications. Unlike natural images, CT images are acquired in controlled environments, resulting in consistent image quality. Consequently, the introduction of degradations such as noise, blur, and JPEG compression, as implemented in Real-ESRGAN, was deemed unnecessary for this application.

Traditional super-resolution networks are typically designed for single-image super-resolution. However, CT images possess volumetric properties, necessitating adaptations to handle 3D data. To accommodate this, we modified the network’s convolutions to process 3D arrays, such as 512 × 512 × 16 arrays. Due to GPU memory constraints, CT volumes were partitioned into segments of 16 slices before being input into the network.

**Figure 2:**
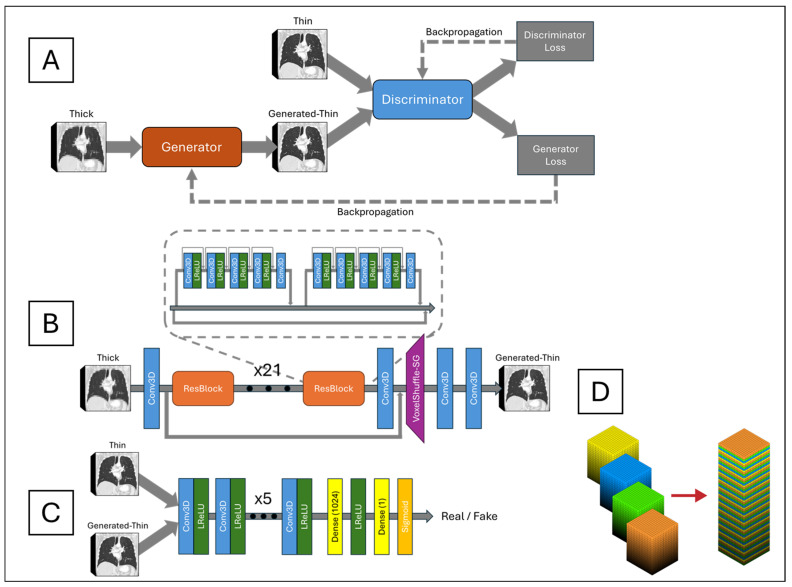
(**A**) Overall network architecture, (**B**) generator model, (**C**) discriminator model, and (**D**) VoxelShuffle-SG.

The network architecture, depicted in Figure 2A, is centered around a generator network, shown in Figure 2B, responsible for converting LR inputs into SR outputs. The generator consisted of 23 ResBlocks as its core building units. Each ResBlock incorporated a central skip connection and comprised two small blocks. Each small block included five alternating 3D CNNs and four leaky rectified linear units (LReLUs), with dense connections preceding each 3D CNN to facilitate residual information flow. Three-dimensional CNN incorporates 64 channels, a kernel size of 3, a stride of 1, and padding of 1. This design improved information propagation efficiency and enhanced the model’s ability to learn complex features. Local residual connections within each small block stabilized the training process by mitigating the vanishing gradient problem commonly observed in deep networks. Additionally, a global residual connection linked the input of the first ResBlock to the output of the last ResBlock, forming a residual-in-residual structure. This dual-level residual architecture enhanced the network’s ability to capture and synthesize high-frequency details critical for reconstructing high-resolution images with fine textures and sharp edges.

Following the ResBlocks’ processing, the network applied a 3D CNN and a custom-designed VoxelShuffle-Slice Generation (SG) module, illustrated in Figure 2D, to enhance spatial resolution in the depth dimension while preserving learned features. The VoxelShuffle-SG module, adapted from PyTorch’s PixelShuffle module [20], was tailored for 3D data processing. Whereas PixelShuffle transforms tensors from the channel domain to the spatial domain in two dimensions, VoxelShuffle-SG extends this functionality to 3D arrays, transferring tensors from the channel domain to the depth dimension. For instance, an input tensor of shape (batch, channel × r^2^, height, width) processed by PixelShuffle resulted in an output tensor of shape (batch, channel, height × r, width × r), where r represents the scaling factor. Similarly, when VoxelShuffle-SG processed an input tensor of shape (batch, channel × r, height, width, depth), the output tensor was reshaped to (batch, channel, height, width, depth × r). Subsequently, two 3D CNN layers generated the final thin-slice CT images.

The generated-thin-slice images were then fed into a discriminator network, shown in Figure 2C, alongside the corresponding ground truth images. The discriminator was designed to distinguish between generated (synthetic) and ground truth (real) thin-slice CT images. Its architecture comprised eight pairs of 3D CNN and LReLU layers, followed by a fully connected dense layer producing 1024 output tensors. These tensors passed through an LReLU activation, followed by another fully connected layer outputting a single value, which was then passed through a sigmoid function. An output of one indicated that the discriminator classified the input as ground truth, while an output of zero indicated that it classified the input as generated. The discriminator was trained adversarially against the generator, driving the generator to produce synthetic thin-slice CT images that became increasingly indistinguishable from the ground truth. Ideally, by the end of training, the generator would produce synthetic images perceptually identical to the ground truth, rendering the discriminator unable to differentiate between the two.

### 2.4. Training

To generate thin-slice from thick-slice CT, first, the image needs to be reformatted in the coronal plane. Due to GPU memory limitations, up to 16 slices of coronal CT images were put into the network for slice generation. Training was conducted using 28,628 chest CT images with an initial slice thickness of 1.0 mm, with each series containing approximately 300 slices on average. To simulate thick-slice images, every four consecutive slices were averaged to produce a single slice, effectively reducing the number of slices per series from 300 to 75 and increasing the slice thickness from 1.0 mm to 4.0 mm. These low-resolution, thick-slice CT images were then input into the network to restore the thin-slice images.

Comparisons between the original 1.0 mm thin-slice CT images and the generated-thin-slice images were performed using three loss functions: mean squared error (MSE) loss (1), perceptual loss (2), and adversarial loss (3). The equations for these loss functions are provided below:

(1) $${\;l}_{n}=\frac{1}{rDHW}\sum_{z=1}^{rD}\sum_{y=1}^{H}\sum_{x=1}^{W}{\left(I_{x,y,z}^{GT}-G_{\theta }{\left(I^{LR}\right)}_{x,y,z}\right)}^{2}$$

(2) $$\;{\;l}_{VGG}^{SR}=\frac{1}{D_{i,j}H_{i,j}W_{i,j}}\sum_{z=1}^{D_{i,\;j}}\sum_{y=1}^{H_{i,j}}\sum_{x=1}^{W_{i,j}}{\left(\varphi_{i,j}{\left(I^{GT}\right)}_{x,y,z}-\varphi_{i,j}{\left(G_{\theta }\left(I^{LR}\right)\right)}_{x,y,z}\right)}^{2}$$

(3) $$l_{Gen}^{SR}=\sum_{n=1}^{N}-logD_{\theta }\left(G_{\theta }\left(I^{LR}\right)\right)$$

where *r* is the scaling factor, which is 4 for this study; *D* is depth; *H* is height; *W* is width; *I^LR^* is a thick-slice CT image; *I^GT^* is a thin-slice CT image; *G_θ_* is the generator function; *D_θ_* is the discriminator function; *φ_i*,*j_* is the feature map obtained after the *j*-th convolution and before the *i*-th max-pooling layer in the VGG network; and *D_i*,*j_*, *H_i*,*j_*, and *W_i*,*j_* are the dimensions of the respective feature map.

The MSE loss is a pixel-wise metric that quantifies the average squared difference between the estimated values and the actual values, effectively measuring the square of the Euclidean distance between them. Although minimizing MSE reduces the overall difference in voxel values between the predicted and actual images, relying solely on MSE can lead to blurry textures due to the suppression of high-frequency details. To mitigate this issue, perceptual and adversarial losses were introduced.

The perceptual loss utilized the VGG16 network, a classification model originally developed for the ImageNet dataset challenge. Let *φ_i*,*j_* represent the feature map obtained after the j-th convolution and before the i-th max-pooling layer in the VGG network, with *D_i*,*j_*, *H_i*,*j_* and *W_i*,*j_* denoting the dimensions of the respective feature map. As the VGG network was designed for natural 2D images, it could not be directly applied to 3D CT images. To address this, the CT volumes were split into individual slices, which were then sequentially fed into the VGG network. The loss was calculated by summing and averaging over all slices. By minimizing the Euclidean distance between the feature representations of the ground truth thin-slice images and the generated-thin-slice images, the network was encouraged to produce images that were perceptually similar.

The adversarial loss was derived from the discriminator in the network, which simultaneously encouraged the generator to produce images more similar to the thin-slice images and trained the discriminator to better distinguish between the generated-thin and thin CT images. This adversarial training was carried out until the loss output saturated in both the training and validation datasets. Upon the completion of training, the model was capable of generating high-quality images that were indistinguishable from the real thin-slice CT images.

### 2.5. Evaluation with AI-Based Lung Cancer Screening Software

**Figure 3:**
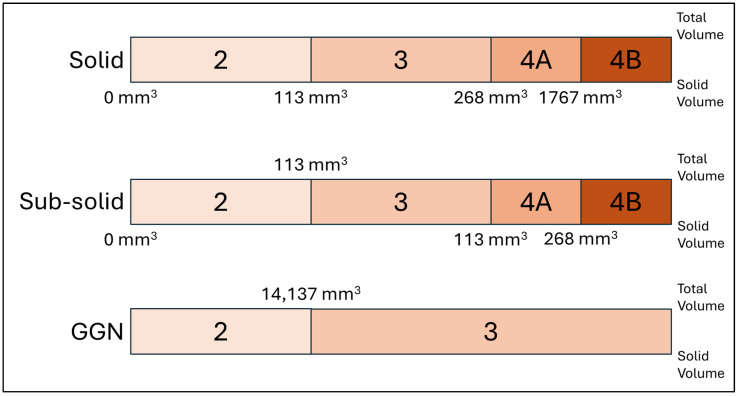
Simplified Lung-RADS v2022 lung nodule categorization criteria for solid, sub-solid, and GGN lung nodules.

**Figure 4:**
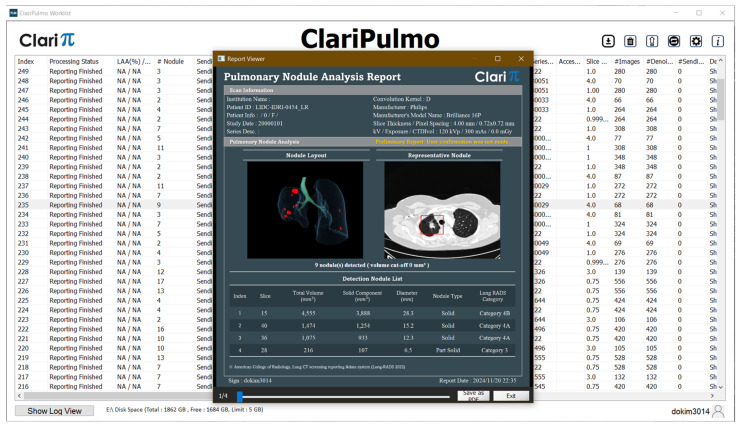
ClariPulmo graphical user interface.

There was a total of 55 pulmonary nodules in the test dataset, and they were presented in the form of thick, generated-thin- and thin-slice CT images. ClariPulmo was used to evaluate their pulmonary nodule volumetry and corresponding pulmonary nodule category. ClariPulmo performs nodule detection, segmentation, and type prediction using deep learning models with CNN architecture and a histogram-based approach for nodule type prediction. The core of the system consists of an end-to-end framework utilizing three modified 3D ResNet architectures for nodule detection and segmentation [21]. These architectures were enhanced with attention mechanisms and weighted loss functions to improve performance. Following nodule detection and segmentation, each nodule was classified into solid, sub-solid, and GGN based on its internal density distribution. This classification used a histogram-based approach, analyzing the distribution of CT attenuation values within each segmented nodule. After that, each type of nodules was sorted into categories 2, 3, 4A, and 4B, in accordance with Lung-RADS v2022, depending on the solid volume and the total volume of the nodule, as illustrated in Figure 3. The software’s graphical user interface is shown on Figure 4.

## 3. Results

**Figure 5:**
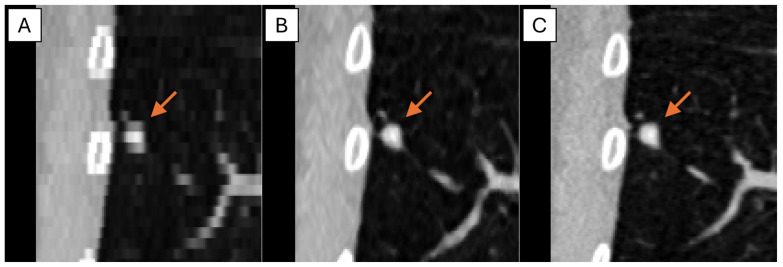
Coronal CT images of (**A**) thick-slice, (**B**) generated-thin-slice, and (**C**) thin-slice presenting solid nodules as shown by the arrows.

**Figure 6:**
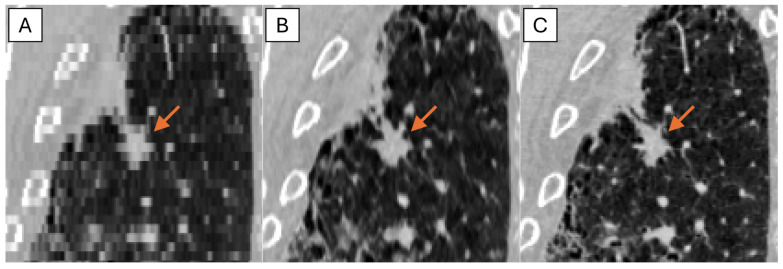
Coronal CT images of (**A**) thick-slice, (**B**) generated-thin-slice, and (**C**) thin-slice presenting GGNs as shown by the arrows.

The representative thick-, generated-thin- and thin-slice coronal CT images of solid nodule and (ground glass nodule) GGN are shown in Figure 5 and Figure 6. The thick-slice CT images show staircase artifacts on both solid nodule and GGN, making accurate volume measurement and corresponding categorization nearly impossible, whereas generated-thin-slice CT images show image quality on par with thin-slice CT images, exhibiting clear nodule/GGN structures and well-defined pulmonary vessels delineations.

**Figure 7:**
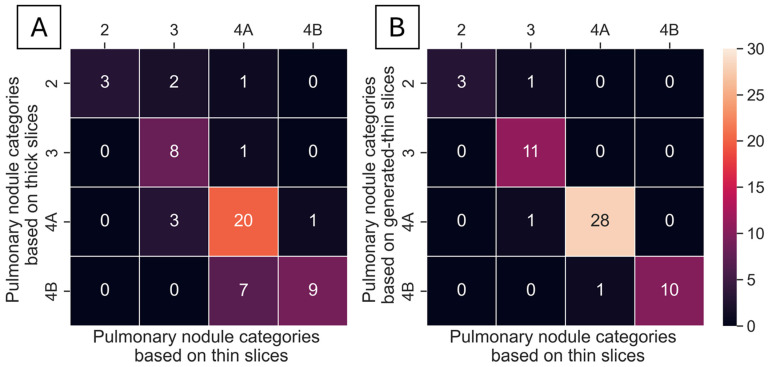
Confusion matrix based on Lung-RADS v2022 lung nodule classification for (**A**) thick-slice–thin-slice CT image pair and (**B**) generated-thin-slice–thin-slice CT image pair.

A total of 55 nodules from 40 series of test datasets were presented in thick-, generated-thin- and thin-slice CT images. These nodules were analyzed using ClariPulmo, and by setting nodule categories from thin-slice CT images as the ground truth, nodule categories from thick-slice and generated-thin-slice CT images were compared using a confusion matrix, as shown in Figure 7. From the confusion matrix, we can see that thick-slice CT images mis-categorized 15 out of 55 nodules, whereas generated-thin-slice CT images mis-categorized 3 out of 55 nodules. Nodule categorization was further analyzed to observe the category accuracy of thick-slice and generated-thin-slice CT images with respect to the ground truth thin-slice CT images. Among 55 nodules, 2 nodules were correctly categorized by thick-slice CT images but incorrectly categorized by generated-thin-slice CT images, 14 nodules were incorrectly categorized by thick-slice CT images but correctly categorized by generated-thin-slice CT images, 1 nodule was incorrectly categorized by both, and 38 nodules were correctly categorized by both. Therefore, thick-slice CT images had a categorization accuracy of 72.7% and generated-thin-slice CT images had a categorization accuracy of 94.5% (Table 2).

## 4. Discussion

The accurate measurement of lung nodule volume is essential for proper categorization in accordance with Lung-RADS v2022. Our results indicate that via using automatic categorization software, lung nodules from thick-slice CT images were misclassified more frequently than those from generated-thin-slice CT images.

CT scans with old CT scanners typically produce images with slice thicknesses between 3 and 5 mm to avoid noise present with thin slices. Even with newer-generation CT scanners, thick-slice images are often stored in Picture Archiving and Communication Systems (PACSs) in preference to thin-slice images for optimizing storage efficiency. In either scenario, the slice thickness is inadequate for AI-based lung cancer screening software to segment and correctly categorize pulmonary nodules present in the chest CT. Similar outcomes were found for attempting to manually segment and correctly categorize pulmonary nodules, which have not been covered in this study, but we have covered them in a separate study, which has been submitted to another journal for a review.

To overcome the slice thickness issue, we implemented a deep learning-enabled super-resolution technique to intake thick-slice CT images and forward pass generated-thin-slice CT images. In detail, we modified our deep learning model based on ESRGAN to accommodate 3D CT images by adjusting the input channels and replacing the PixelShuffle module with the VoxelShuffle-SG module, which enabled the model to generate thin-slice CT images and recover both nodule volume and texture faithfully. Therefore, thick-slice CT images can be converted to generated-thin-slice CT images and produce accurate categorization of pulmonary nodules by ClariPulmo. This study showed that the automatic nodule mis-categorization decreased from 15 cases down to merely 3 cases out of 55 cases examined when thick-slice CT images were converted to generated-thin-slice CT images. The outcome indicated that applying super-resolution-based slice generation to thick-slice CT images prior to automatic pulmonary nodule categorization significantly increased the categorization accuracy with little to no side-effects.

This study has certain limitations. Although training was conducted using data from four major CT vendors (Siemens Healthineers, GE Healthcare, Canon Medical Systems, and Philips Healthcare), all images were sourced from the NLST open dataset. Similarly, the test data originated from the LIDC-IDRI open dataset. Future work should include validating the model’s performance on CT images from other institutions and with various CT scanners to ensure generalizability.

## 5. Conclusions

This study aimed to address the challenges posed by heterogeneous slice thickness in CT imaging, which can negatively impact the accuracy of automated pulmonary nodule categorization. To mitigate this issue, we employed a deep learning-enabled 3D super-resolution method to convert thick-slice CT images into uniform, generated-thin-slice CT images. The effectiveness of this approach was evaluated by comparing pulmonary nodule categorization outcomes from both thick-slice and generated-thin-slice CT images using AI-based lung cancer screening software, with reference thin-slice CT images serving as the gold standard. The results demonstrated a notable improvement in categorization accuracy, with thick-slice CT images achieving 72.7% accuracy, while the generated-thin-slice CT images attained a higher accuracy of 94.5%. These findings underscore the potential of our proposed approach to enhance diagnostic precision in scenarios where only thick-slice CT images are available. In conclusion, the conversion of thick-slice CT images into generated-thin-slice CT images represents a promising strategy for improving the reliability of pulmonary nodule categorization.

## Figures and Tables

**Table 1:** Parameters of training datasets.

Manufacturer	Siemens Healthineers	Canon Medical Systems	GE Healthcare	Philips Healthcare
Model	Sensation 16	Aquilion	LightSpeed 16	MX8000
Tube Voltage (kV)	120	120	120	120
Slice Thickness (mm)	1.0	1.0	1.0	1.0
Reconstruction Kernel	B30f, B45f, B50f, B80f	FC01, FC02, FC53	Standard, Lung	C
Number of Slices	18,243	2931	2707	4747

**Table 2:** Categorization outcomes using ClariPulmo.

Thick Correct/ Generated-Thin Incorrect	Thick Incorrect/Generated-Thin Correct	Thick Incorrect/Generated-Thin Incorrect	Thick Correct/Generated-Thin Correct	Thick-Slice Categorization Accuracy	Generated-Thin-Slice Categorization Accuracy
2	14	1	38	72.7%	94.5%

## Data Availability

The LIDC-IDRI CT image dataset used for the evaluation is available at the following URL: https://www.cancerimagingarchive.net/collection/lidc-idri/, accessed on 10 September 2024. The NLST CT image dataset used for training the model is available at the following URL: https://www.cancerimagingarchive.net/collection/nlst/, accessed on 2 June 2024.
